# Sports-Related Pure Orbital Blowout Fractures in Japan: Differences in Demographic and Clinical Characteristics between Sports

**DOI:** 10.3390/diagnostics14090913

**Published:** 2024-04-27

**Authors:** Jose Miguel Ambat, Steffani Krista Someda, Hirohiko Kakizaki, Yasuhiro Takahashi

**Affiliations:** Department of Oculoplastic, Orbital & Lacrimal Surgery, Aichi Medical University Hospital, Nagakute 480-1195, Aichi, Japan; josemiguelambat@gmail.com (J.M.A.); steffsomeda@gmail.com (S.K.S.); cosme_geka@yahoo.co.jp (H.K.)

**Keywords:** sports-related fracture, pure orbital blowout fracture, binocular single vision, fracture pattern, ocular and periocular injuries

## Abstract

The aim of this study was to present the demographic and clinical characteristics of sports-related pure (rim-sparing) orbital blowout fractures and to analyze these differences by type of sport. Ten years of sports-related orbital fracture data were accumulated. Patients were classified into similar sports (i.e., soccer and futsal) wherein orbital blowout fractures were obtained, producing 14 groups. This study included 377 sides from 374 patients. The majority of patients were male (83.4%), and the mean population age was 20.9 ± 10.8 years. The most common sports causing injuries were baseball/softball, rugby/football, and martial arts. Single-wall fractures were found in 78.8% of patients, but baseball/softball had a higher frequency of multiple-wall fractures (*p* = 0.035). Concomitant ocular and periocular injuries occurred in 18.6% of patients, which were frequently caused by baseball/softball (*p* < 0.001). The field of binocular single vision (BSV) included primary gaze in 84.2% of patients. Surgical management was conducted in 52.1% of patients. This study showed that baseball and softball had the highest rate of multi-wall fractures and concomitant ocular and periocular injuries. The field of BSV measured during the first examination was acceptable in most cases.

## 1. Introduction

Orbital blowout fractures are a frequently encountered condition by orbit and oculoplastic surgeons [1]. These originate from blunt force energy transmission to the orbital walls, either directly from the orbital rim (buckling theory) or indirectly from increased intraorbital pressure (hydraulic theory) [1,2]. Trauma etiologies differ between age groups but can generally be categorized into falls, interpersonal violence, motor vehicular accidents, and sports [3]. The resulting blowout fractures can be further classified into either pure or impure fractures: pure orbital blowout fractures have an intact orbital rim, while impure orbital blowout fractures have associated rim fractures [1].

Orbital fractures account for 5.0 to 33.5% of sports-related maxillofacial fractures [4,5,6,7,8,9]. Previous studies seldom document if these are blowout fractures, and if so, whether they are pure (rim-sparing) or impure (rim involvement). Lock et al. previously reported on sports-related orbital fractures, documenting the prevalence rate at 13.5%, noting the most common sports to cause injury as soccer, bicycling, and jogging [8]. However, regularly played sports differ between countries, and international differences in culture and climate inevitably lead to varied preferences in sports [7]. For example, the primary sports that cause maxillofacial and ocular injuries are soccer in Australia and the Netherlands, Gaelic football in Ireland, ice hockey in Finland, and skiing in America [4,5,6,7,9]. Unique demographics and play styles exist within each sport, which may modify the characteristics of pure orbital blowout fractures obtained during play.

Japan, a mountainous archipelago with four seasons and historical ties to Eastern and Western cultures, is no different. The top spectator sports in Japan, as of 2023, in order, were baseball, football (soccer), basketball, volleyball, martial arts, and rugby [10]. Japan also tends to have high rates of physical activity. The survey by the Agency for Cultural Affairs in 2020 showed that 67.1% of middle and high school students participate in after-school sports club activities [11]. In addition, it has been documented that 24.8% of adult men and 22.9% of adult women participate in moderate-intensity physical activities at least twice a week for 30 min [10].

The purpose of this study is to present the demographics, clinical characteristics, and associated features of pure orbital blowout fractures among the different sports commonly played in Japan. Data from the present study will contribute to the body of knowledge on sports-related pure orbital blowout fractures.

## 2. Materials and Methods

This was a retrospective study including all patients with orbital fractures referred to our service from May 2013 to December 2023. Our hospital introduced an electronic medical chart system in May 2013; hence, data retrieval commenced from this date. Patients with sports-related pure orbital blowout fractures were included in this study. Patients with concomitant orbital-rim fractures, i.e., impure orbital fractures, and those with old orbital fractures, were excluded. Patients in whom the orbital fractures were not directly acquired while playing sports (e.g., falling down while playing golf) were also excluded.

The data on age; sex; laterality; causative sports; concomitant ocular and periocular injuries; fields of binocular single vision (BSV) examined on the initial visit; and surgery were collected. Similar sports were grouped (e.g., soccer and futsal), resulting in 14 categories of causative sports (Table 1). The results for the field of BSV were classified into five categories according to our previous study: B1—the field of BSV is within the normal range in the Japanese population (±2 × standard deviation); B2—the field of BSV reaches at least 20 degrees superiorly, 40 degrees inferiorly, and 30 degrees horizontally; B3—a smaller field of BSV than B2 but includes primary gaze; B4—the field of BSV does not include primary gaze; and B5—cannot obtain the field of BSV in any direction of gaze [12]. Data on the presence of enophthalmos were not collected because orbital soft-tissue edema, caused by trauma, prevents accurate Hertel exophthalmometric measurements.

We strongly recommended surgical reduction to patients when the field of BSV was B3 or worse. Additional indications for surgical reduction included: (1) young patients, even with a BSV grade of B2, since higher physical activity demands a wider field of BSV, and (2) patients with a large medial orbital-wall fracture to prevent enophthalmos.

**Figure 1:**
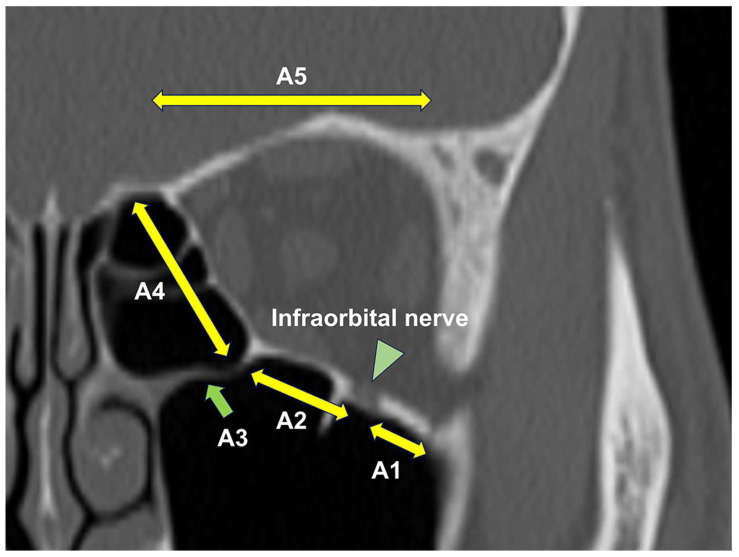
Classification of orbital fracture sites. A1, the orbital floor lateral to the infraorbital nerve (green arrowhead); A2, the orbital floor medial to the infraorbital nerve (green arrowhead); A3, the inferomedial orbital strut (green arrow); A4, the medial orbital wall; A5, the orbital roof.

Axial and coronal computed tomographic (CT) images with bone and soft-tissue window algorithms were obtained from all patients. Orbital fracture sites, entrapped orbital soft tissues in cases with trapdoor fractures, and concomitant nasal bone fractures were examined. Orbital fracture sites were classified into A1-A5 based on previous studies (Figure 1) [3].

Patients were grouped according to causative sports. Patient age was expressed as means ± standard deviations. Population age was compared among groups using one-way ANOVA and Tukey’s post hoc test. A chi-square test was employed to compare the categorical variables among groups. All statistical analyses were performed using SPSS™ version 26 software (IBM Japan, Tokyo, Japan). Two-tailed *p* values < 0.05, with a 95% confidence interval, were deemed to indicate statistical significance.

## 3. Results

Data on patient characteristics are shown in Table 1. Among 1171 sides from 1152 patients with pure orbital blowout fractures, 377 sides (32.2%) from 374 patients were included in this study. The study population consisted of 312 males (83.4%) and 62 females (16.6%). Patient age was 20.9 ± 10.8 years. The most common causative sports, in order, were baseball/softball (132 patients/sides), followed by rugby/American football (64 sides from 62 patients), and martial arts, including mixed martial arts, boxing, kick boxing, wrestling, karate, judo, and sumo wrestling (53 sides from 52 patients).

Data on clinical findings are shown in Table 2. Ocular and periocular injuries were documented in 70 sides (18.6%) from 70 patients. The most common injuries were commotio retinae (33 patients) and hyphema (22 patients). The field of BSV included primary position (B3 or better) in 315 patients (84.2%). Orbital fractures were reduced in 195 patients (52.1%).

Data on radiological findings are shown in Table 3. Fractures were limited to the orbital floor or medial orbital wall (single-wall fracture) in 297 sides (78.8%), while fractures extended to both the orbital floor and medial orbital wall in 80 sides (21.2%). The inferomedial orbital strut and orbital roof were fractured on 23 sides (6.1%) and 3 sides (0.8%), respectively. Trapdoor fractures were documented in 144 sides (38.2%), among which the extraocular muscle was incarcerated in 16 sides (4.2%). The nasal bone was concomitantly fractured in 16 patients (4.2%).

The results of the statistical comparison for each item among the patient groups are shown in Table 4. Although most groups predominantly included male patients, a substantial number of female patients were included in the groups for basketball, skiing/snowboarding, volleyball, and lacrosse. The male-to-female ratio was significantly different among the groups (*p* < 0.001). The laterality of the injured orbit was not significantly different among the groups (*p* = 0.656). Patients who performed dodgeball and apparatus gymnastics, floor exercises, or cheerleading were relatively younger, while patients who engaged in skiing/snowboarding, surfing, or weight training were comparatively older (*p* < 0.001). Both the orbital floor and medial orbital wall were more frequently fractured by baseball/softball injuries (*p* = 0.035). The proportions of patients with inferomedial orbital strut fractures (*p* = 0.624) or trapdoor fractures (*p* = 0.301) were not different among the groups. Approximately 3/4 of patients with ocular and periocular injuries (54 of 70 patients) were injured while playing baseball/softball (*p* < 0.001). Nasal bone fractures were largely associated with martial arts and weight training (*p* = 0.030). The distribution of the field of BSV was different among the groups (*p* = 0.012), and accordingly, the proportion of patients who underwent surgical reduction tended to be different among the groups (*p* = 0.050).

## 4. Discussion

The current investigation presented the demographics and clinical characteristics of sports-related pure orbital blowout fractures and analyzed these differences by type of sport. The patient population was mostly male, with a mean age of 20.9 ± 10.8 years. Groups 1 (baseball/softball), 2 (rugby/American football), and 3 (martial arts) were the primary causative sports. Single-wall fractures were frequently documented with a concomitant injury rate of 18.6%. Of the 374 patients, 315 (84.2%) cases had a field of BSV including the primary position of gaze. Surgical reduction was performed in 195 of 374 patients (52.1%).

The overall study population was predominantly male—312 out of 374 patients (83.4%). Sex-related differences between groups were likewise significant (*p* < 0.001), with only groups 6 (basketball), 8 (handball), 10 (volleyball), and 12 (lacrosse) having a considerable number of female patients. Male predominance in sports-related maxillofacial and orbital fractures has been well documented, with incidence rates ranging from 77.4% to 94.7% [8,13]. Reasons for male proclivity are multifactorial. These include gender stereotypes concerning what constitutes masculine and feminine sports and the pressure to conform to societal gender norms [14]. Masculine sports are stereotypically associated with physical contact, face-to-face opposition, and aggressiveness, all of which may increase the risk of sustaining injury [14].

Single-wall fractures were prominent in the overall study population (78.8%) and among groups (*p* = 0.035). Preceding studies have determined that sports-related maxillofacial and orbital injuries generally occur as a result of the following: falls during play, contact with other players, contact with equipment or projectiles (e.g., ball pitches, golf clubs), and collision with the environment (e.g., colliding with a tree) [4,5,7,9,15,16,17,18]. The present study excluded falls; thus, only contact with players, equipment, and the environment was included. Contact between players was the leading cause of injury in five out of eight studies once falling was disregarded [4,5,7,9,15,16,17,18]. The single-wall fracture prominence among patient groups suggests that causes of orbital blowout fractures generate low-energy forces. This theory is supported by the results of group 3, which are typically identified as contact sports, and the low incidence of orbital roof fractures. Although striking and intentional physical contact are inherent in martial arts, 48 out of 53 (90.6%) of group 3 fractures involved a single orbital wall. Contact between players in group 3, and the other groups, may be classified as assault or interpersonal violence as the mechanism of injury is similar. Assault produces low-energy forces when compared to other etiologies of orbital blowout fractures [19,20]. When comparing motor vehicular accidents (MVAs) to interpersonal violence, MVAs exhibited more complex injuries, a higher Le Fort fracture incidence (13% vs. 1.6%), and higher hospitalization rates (87% vs. 59%) [20]. Lastly, the incidence rate of orbital roof fractures in the study was <1.0% (*n* = 3). Orbital roof fractures are associated with high energy forces, and their presence raises the incidence of concomitant ocular and periocular injuries [21,22].

Group 1 had the highest rate of multiple orbital-wall fractures at 33.3%, which was significant (*p* = 0.035). Baseball and softball are non-contact sports, with the major cause of injury coming from contact with the ball (46.1–70.3%) [9,23]. Despite baseballs used by elementary and junior high school students in Japan being softer than their Western counterparts, ball velocities can range from 95.2 to 160.9 km/h, generating enough energy to involve multiple orbital walls [24,25]. The present study findings corroborate preceding investigations conducted in Korea and America, which documented that baseball and softball account for 10.5–26.9% of all maxillofacial injuries and commonly incur multiple orbital-wall fractures [13,23,26]. Increased morbidity is also associated with group 1 as 54 out of 132 (40.9%) patients and 70 (77.1%) of associated ocular and periocular injuries were due to baseball and softball (*p* < 0.001). Two out of three cases of orbital roof fractures and the sole cases of globe rupture and nasolacrimal canal fracture were also documented in group 1.

Trapdoor fractures were documented in 144 affected sides (38.2%) within the overall study population. Despite the results being statistically insignificant among groups (*p* = 0.301), trapdoor fractures occurred most in group 4 wherein patients were the youngest. Children are known to have elastic bones due to increased amounts of cancellous bone and fewer cortices [3]. Due to its pliable nature, the hinged type of blowout fractures snaps back into position quickly, trapping a considerable amount of tissue [2,27]. This elasticity is slowly lost as the patient ages, hence the lower rate of trapdoor fractures in adults and inversely higher rates in the young [3,27].

Concomitant ocular and periocular injuries were noted in 70 (18.6%) sides. The most common injuries were commotio retinae (*n* = 33) and hyphema (*n* = 22). Injury overlap exists between patients, but more posterior segment injuries (*n* = 54) were documented than anterior segment injuries (*n* = 36). Contrecoup force transmission to the posterior of the globe, via the hydraulic theory, explains this as well, as posterior segment structures are less pliable than anterior segment structures [2,28].

Nasal bone fractures were significantly associated with group 3 and group 11 (*p* = 0.030). Martial arts, which primarily include striking, are known to target the head, with the middle-third of the face accounting for the bulk of maxillofacial trauma in the related literature (49.5%) [29,30]. The overall etiology of nasal fractures cites interpersonal violence as the top cause, at 35.8% [31]. The literature for group 11-related orbital trauma is sparse. Türegün et al. reported two incidences wherein weights fell on the middle-third of the face during weight training, causing nasal fractures; both cases were due to absent or inattentive spotters [32]. This is similar to the present study as patients in group 11 had weights fall on the middle-third of the face while performing a bench press.

The field of BSV included the primary position of gaze in 315 (84.2%) patients. Differences in BSV were statistically significant between groups (*p* = 0.012). In our institution, the BSV test is used, alongside the Hess chart, to assess if a patient is a surgical candidate. The present study advised surgical reduction to younger patients even with a BSV score of B2 to increase the field of vision and prevent diplopia post-reduction. Urgent surgical reduction has been documented to improve post-operative motility and field of vision better in younger patients than adults [27,33].

Surgical intervention was performed in 195 (52.1%) patients. Differences in the surgical reduction rate were statistically significant among groups (*p* = 0.050). The reduction of fractures was less than 50% in groups 1, 5 (soccer, futsal), and 7. Despite group 1 having the greatest number of multiple-wall fractures and concomitant ocular injuries, surgical reduction was performed in only 57 of 132 patients (43.2%), which was low compared to the other groups. This is explained by different variables, which include the patient’s age at the time of the incident, the desire for surgery, and visual acuity. The number of patients with unmeasurable BSV and B5 was highest in group 1. The poor prognosis of the visual outcome, due to the higher incidence of posterior segment injuries and the ruptured globe, may have decreased the number of potential surgical candidates.

This study has several limitations. First, the incidence rate of orbital blowout fractures may not have high external validity. Orbital blowout fractures with an etiology of falling were excluded in this study. Since falling is frequently included in the top three causes of orbital blowout fractures, the incidence rate may be higher than that reported in the present investigation [2,3,9,15,16,34,35]. Similarly, patient age affects the incidence rate of orbital blowout fractures as children are known to have steeper orbital floors and are thus more prone to orbital blowout fractures [3]. The stratification of similar sports into groups also influences the incidence rate as styles of play and participant demographics are different between sports. Second, ethnicity may play a role in the incidence of orbital blowout fractures as all patients included are of Japanese descent. It should be stated that a previous study documented insignificant variation in orbital blowout fracture rates between different ethnicities [36]. The study, however, grouped patients from India and eastern Asia together as a homogenous group of Asians and therefore cannot be used to generalize for the entire Asian population [36].

## 5. Conclusions

Sports-related pure orbital blowout fractures frequently involve male patients (83.4%). The study population was relatively young, with a mean age of 20.9 ± 10.8 years. The most common sports causing injuries were baseball/softball, rugby/football, and martial arts. A predominance of single-wall fractures was observed; however, particular sports had higher rates of multiple-wall fractures. Ocular and periocular injury rates were highest in group 1, while concomitant nasal bone fractures were highest in groups 3 and 11. The field of BSV included primary gaze in 84.2% of patients, with a total surgical reduction rate of 52.1%. Surgery is advised, especially in younger patients, to preserve the field of vision and prevent enophthalmos and diplopia.

## Figures and Tables

**Table 1:** Demographic data.

Number of patients/sides	374/377
M/F	312/62
R/L	155/222
Age (years)	20.9 ± 10.8
Causative sports (sides)	
1. Baseball/softball	132
2. Rugby/American football	64 (bilateral in 2 cases)
3. Martial arts including MMA, boxing, kick boxing, wrestling, karate, judo, and sumo wrestling	53 (bilateral in 1 case)
4. Apparatus gymnastics/floor exercises/cheerleading	33
5. Soccer/futsal	29
6. Basketball	20
7. Skiing/snowboarding	12
8. Handball	7
9. Surfing	6
10. Volleyball	6
11. Weight training	5
12. Lacrosse	3
13. Dodgeball	2
14. Others	5

M, male; F, female; R, right; L, left; MMA, mixed martial arts. “Others” include cases with golf-, horse-riding-, skateboarding-, tennis-, and badminton-related fractures.

**Table 2:** Clinical data.

Ocular/periocular injury ^1^ (patients/sides)	70/70
Commotio retinae	33
Hyphema	22
Mydriasis	7
Iritis	5
Macular hole	5
Vitreous hemorrhage	5
Retinal tear	3
Lacrimal canalicular laceration	2
Maculopathy	2
Orbital hematoma	2
Retinal hemorrhage	2
Serous macular detachment	2
Traumatic ptosis	2
Iridodialysis	1
Lens subluxation	1
Retinal detachment	1
Choroidal hemorrhage	1
Globe rupture	1
Eyelid laceration	1
Bony nasolacrimal canal fracture	1
Orbital compartment syndrome	1
Preoperative field of BSV ^2^	
B1	105
B2	113
B3	97
B4	20
B5	24
Unmeasurable	15
Surgical reduction	195

^1^ Multiple injuries are present in select patients, hence the greater number of injuries compared to the number of patients and sides involved. ^2^ BSV, binocular single vision.

**Table 3:** Radiographic findings.

Fracture Sites	
Single-wall fracture (including A1 + A2)	297
Multiple-wall fracture	80
Strut fracture	23
Orbital roof fracture	3
Trapdoor fracture	144
Extraocular muscle incarceration	16
Concomitant nasal bone fracture	16

**Table 4:** Data in each group and results of statistical comparison.

Group Number	1	2	3	4	5	6	7
Number of patients/sides	132/132	62/64	52/53	33/33	29/29	20/20	12/12
M	107 (81.1%)	61 (98.4%)	51 (98.1%)	23 (69.7%)	27 (93.1%)	11 (55.0%)	10 (83.3%)
F	25 (18.9%)	1 (1.6%)	1 (1.9%)	10 (30.3%)	2 (6.9%)	9 (45.0%)	2 (16.7%)
R	54 (40.9%)	28 (43.8%)	16 (30.2%)	18 (54.5%)	11 (37.9%)	9 (45.0%)	5 (41.7%)
L	78 (59.1%)	36 (56.3%)	37 (69.8%)	15 (45.5%)	18 (62.1%)	11 (55.0%)	7 (58.3%)
Age (years)	20.7 ± 11.5	19.6 ± 2.0	20.7 ± 6.5	13.9 ± 4.8	18.4 ± 7.2	18.7 ± 7.9	42.5 ± 11.3
Single-wall fracture	88 (66.7%)	51 (79.7%)	48 (90.6%)	26 (78.8%)	26 (89.7%)	18 (90.0%)	10 (83.3%)
Multiple-wall fracture	44 (33.3%)	13 (20.3%)	5 (9.4%)	7 (21.2%)	3 (10.3%)	2 (10.0%)	2 (16.7%)
Strut fracture	13 (9.8%)	4 (6.3%)	1 (1.9%)	3 (9.1%)	0 (0.0%)	1 (5.0%)	0 (0.0%)
Trapdoor fracture	47 (35.6%)	24 (37.5%)	24 (45.3%)	18 (54.5%)	10 (34.5%)	8 (40.0%)	2 (16.7%)
Extraocular muscle incarceration	3 (2.3%)	1 (1.6%)	4 (7.5%)	1 (3.0%)	1 (3.4%)	2 (10.0%)	0 (0.0%)
Ocular/periocular injury	54 (40.9%)	3 (4.7%)	3 (5.7%)	1 (3.0%)	4 (13.8%)	1 (5.0%)	0 (0.0%)
Concomitant nasal bone fracture	4 (3.0%)	2 (3.2%)	5 (9.6%)	1 (3.0%)	2 (6.9%)	0 (0.0%)	0 (0.0%)
Field of BSV							
B1	49 (39.2%)	16 (26.7%)	9 (18.0%)	7 (22.6%)	10 (35.7%)	4 (20.0%)	2 (16.7%)
B2	39 (31.2%)	19 (31.7%)	13 (26.0%)	12 (38.7%)	8 (28.6%)	7 (35.0%)	6 (50.0%)
B3	23 (18.4%)	22 (36.7%)	15 (30.0%)	11 (35.5%)	9 (32.1%)	4 (20.0%)	4 (33.3%)
B4	5 (4.0%)	2 (3.3%)	6 (12.0%)	0 (0.0%)	0 (0.0%)	2 (10.0%)	0 (0.0%)
B5	9 (7.2%)	1 (1.6%)	7 (14.0%)	1 (3.2%)	1 (3.6%)	3 (15.0%)	0 (0.0%)
Unmeasurable	7	2	2	2	1	0	0
Surgical reduction	57 (43.2%)	35 (56.5%)	35 (67.3%)	19 (57.6%)	14 (48.3%)	13 (65.0%)	4 (33.3%)
**Group Number**	**8**	**9**	**10**	**11**	**12**	**13**	***p* value**
Number of patients/sides	7	6	6	5	3	2	
M	4 (57.1%)	6 (100%)	1 (16.7%)	5 (100%)	1 (33.3%)	2 (100%)	<0.001
F	3 (42.9%)	0 (0.0%)	5 (83.3%)	0 (0.0%)	2 (66.7%)	0 (0.0%)	
R	1 (14.3%)	2 (33.3%)	3 (50.0%)	3 (60.0%)	2 (66.7%)	1 (50.0%)	0.656
L	6 (85.7%)	4 (66.7%)	3 (50.0%)	2 (40.0%)	1 (33.3%)	1 (50.0%)	
Age (years)	17.4 ± 3.5	39.2 ± 9.4	24.5 ± 17.1	37.8 ± 19.6	20.3 ± 1.2	6.5 ± 3.5	<0.001
Single-wall fracture	6 (85.7%)	6 (100%)	5 (83.3%)	4 (80.0%)	2 (66.7%)	2 (100%)	0.035
Multiple-wall fracture	1 (14.3%)	0 (0.0%)	1 (16.7%)	1 (20.0%)	1 (33.3%)	0 (0.0%)	
Strut fracture	1 (14.3%)	0 (0.0%)	0 (0.0%)	0 (0.0%)	0 (0.0%)	0 (0.0%)	0.624
Trapdoor fracture	2 (28.6%)	0 (0.0%)	3 (50.0%)	1 (20.0%)	2 (66.7%)	1 (50.0%)	0.301
Extraocular muscle incarceration	0 (0.0%)	0 (0.0%)	2 (33.3)	1 (20.0%)	0 (0.0%)	1 (50.0%)	
Ocular/periocular injury	0 (0.0%)	1 (16.7%)	1 (16.7%)	0 (0.0%)	0 (0.0%)	0 (0.0%)	<0.001
Concomitant nasal bone fracture	0 (0.0%)	0 (0.0%)	0 (0.0%)	2 (40.0%)	0 (0.0%)	0 (0.0%)	0.030
Field of BSV							
B1	1 (14.3%)	0 (0.0%)	2 (33.3%)	1 (20.0%)	1 (33.3%)	0 (0.0%)	0.012
B2	2 (28.6%)	1 (20.0%)	0 (0.0%)	2 (40.0%)	2 (66.7%)	1 (50.0%)	
B3	4 (57.1%)	3 (60.0%)	1 (16.7%)	1 (20.0%)	0 (0.0%)	0 (0.0%)	
B4	0 (0.0%)	1 (20.0%)	2 (33.3%)	1 (20.0%)	0 (0.0%)	0 (0.0%)	
B5	0 (0.0%)	0 (0.0%)	1 (16.7%)	0 (0.0%)	0 (0.0%)	1 (50.0%)	
Unmeasurable	0	1	0	0	0	0	
Surgical reduction	6 (85.7%)	3 (50.0%)	5 (83.3%)	3 (60.0%)	0 (0.0%)	1 (50.0%)	0.050

Group numbers correspond to the numbers of causative sports shown in Table 1. M, male; F, female; R, right; L, left; BSV, binocular single vision.

## Data Availability

Data supporting the results of this study are available upon request.
